# Investigation of the evaporation behavior of aroma compounds in e-cigarettes

**DOI:** 10.1007/s00216-019-01749-7

**Published:** 2019-03-16

**Authors:** Jean-Christophe Noël, Veronika Ruzsanyi, Matthias Rainer, Günther Bonn

**Affiliations:** 1 Institute of Analytical Chemistry and Radiochemistry, University of Innsbruck, CCB- Center of Chemistry and Biomedicine, Innrain 80-82, 6020 Innsbruck, Austria; 2 Austrian Drug Screening Institute, Innrain 66a, 6020 Innsbruck, Austria; 3 Institute for Breath Research, University of Innsbruck, 6850 Dornbirn, Austria

**Keywords:** Benzaldehyde, Estragole, Limonene, e-cigarette flavor

## Abstract

The aim of this work was to evaluate the evaporation behavior of certain aroma compounds found in e-liquids. Since an e-liquid is evaporated, the aroma present can reach the lungs and could be absorbed into the body which may have long-term health effects above critical concentrations. Due to a lack in regulations, the sort and concentration of the compounds in sold e-liquids can vary. To capture the aroma compounds in the vapor, a smoking machine was developed. The resulting data represent the amount of aroma reaching the consumers’ lungs. The influence of the e-cigarette temperature, ranging from 100 to 315 °C, on the evaporation of benzaldehyde, estragole, and different terpenoids was examined. Additionally, the effect of the liquid base composition on the amount of aroma in the vapor was compared using the analysis of variances. The influence of high temperature, the type of e-cigarette, and the atomizer coil material, which could lead to oxidation of limonene and linalool in the vapor, is shown here.

**Electronic supplementary material:**

The online version of this article (10.1007/s00216-019-01749-7) contains supplementary material, which is available to authorized users.

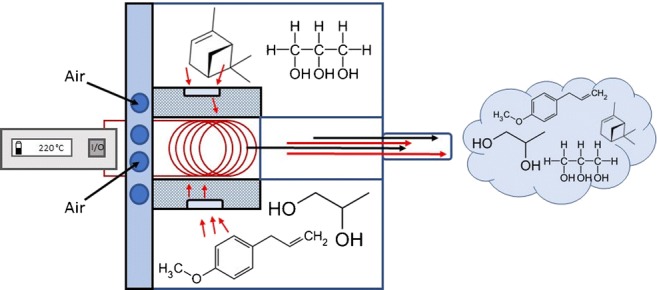

## Introduction

More and more consumers are using electronic cigarettes as an alternative nicotine delivery system. The e-liquid base used in these cigarettes consists of mainly propylene glycol and glycerin. These two compounds form an aerosol through the heated element—the atomizer—in the e-cigarette. Nicotine and aroma compounds are added to this base, the latter influencing the smell of the produced vapor. Different kinds of flavors are available to the consumer, ranging from a typical fruit aroma up to strange ones such as salami pizza. The big variety of flavors is attractive to young e-cigarette users, who are not primary interested in nicotine intake but the smell [1]. Given the increase in the use of e-cigarettes, it is surprising that neither the composition nor the concentration of different aroma compounds is regulated. The EU guidelines for tobacco products do not mention anything about the composition of the e-liquid, but only lists prohibited compounds in tobacco products, including vitamins, cooling agents, and caffeine [2]. The difficulty lies in the contentious issue as to if e-liquids can be viewed as a tobacco product or food product. The EU regulations for aroma used in food by the European Union only manage the concentration of certain possible toxic compounds in food [3]. The quantification of certain possible toxic aroma compounds in e-liquids was done elsewhere [4]; for example, Vardavas et al. found in average 0.0268% (m/m) of linalool in 31.1% studied e-liquid samples [5]. Clapp et al. quantified cinnamaldehyde in the vapor, ranging between 0.742 and 187.9 mM [6]. Estragol was only identified by Peace et al. in one e-cigarette vapor [7]. Kosmider et al. showed that benzaldehyde is mainly emitted when using cherry flavored e-liquids with concentrations between 5.12 and 141.2 μg/30 puffs [8]. The here tested aroma compounds were selected due to their boiling point, polarity, and, in the case of benzaldehyde and estragole, their potential toxicity. A major goal of this paper is to report this together with their evaporation behavior as a function of operating temperature and liquid base composition.

Different brands of e-cigarettes run at different temperatures with a maximum of about 315 °C. At such a high-temperature oxidation of various terpenoids, such as limonene or linalool, can take place, and hence, this needs to be investigated. Both compounds might form skin irritating limonene oxides or linalool peroxides [9–11]. The most crucial part of evaporation behavior is the used smoking machine. It has to be quantitative for the capturing process and reproducible. By comparing the results of different compounds emitted by an e-cigarette, the coefficient of variation (CV) can be used as an indicator for reproducibility of experiments in the present literature. Here, the error consists of the error of the pump, the e-cigarette evaporation error, the solubility of the vapor in the solvent, and the error of the GC/MS. When the literature for analysis of compounds evaporated from or produced in e-cigarettes is compared, most of the coefficients or variations lie above 20%. Goniewicz et al. achieved an average CV of 15–23% for nicotine by using 300 puffs on a self-made smoking machine [12]. Whereas Geiss et al. achieved a CV of 5% for nicotine and 6–15% for the liquid base using a professional Borgwaltd smoking machine [13]. If the formation of carbonyls is analyzed, the average CV is above 20%, which could be due to either the used smoking machine or the analytical method [14–16]. The goal of the smoking machine used in this publication was the increase in reproducibility by using inexpensive parts.

## Material and methods

### Chemicals

Benzaldehyde (99.5%), estragole (98%), t-anethole (99%), R-limonene (99%), α-pinene (99%), β-pinene (99%), linalool (97%), rac.-limonene oxide (97%), rac.-linalool oxide (97%), 1,8-cineole (99%), α-terpinene (95%), propylene glycol (99.5%), glycerin (99.5%), acetonitrile (HPLC grade), p-cymene (99%), rac. L-carveol (95%), and R-carvone (98%) were purchased by Sigma-Aldrich.

### Establishment of a smoking machine

**Fig. 1:**
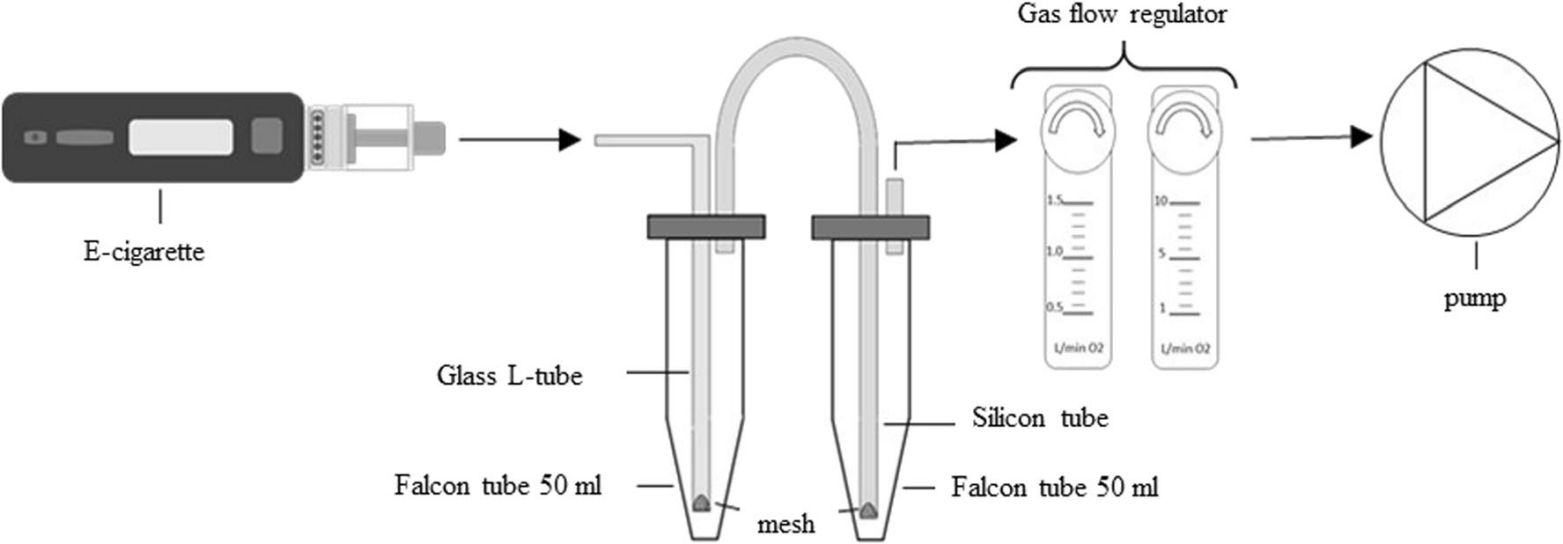
Scheme of smoking machine applied for e-cigarette vapor analysis

Figure 1 shows the used vapor capturing device. Two 50-ml Falcon tubes were connected to each other via a silicon tube that penetrated their caps. The e-cigarette was connected to the first falcon with an L-shaped glass tube. Each end of the tube which was inserted into the washing solution was equipped with a small metal mesh (Gerstel SS Screens). The second falcon tube was connected to a gas flow regulator (MagiDeal acryl gas air oxygen flowmeter) and a gas pump (Carl Roth dry vacuum pump). The e-cigarette and pump were activated by hand. Each falcon tube was filled with 4 mL of acetonitrile. The flowmeter and the flow of the smoking machine were adjusted by attaching a 250-mL gas-tight syringe (Socorex, Switzerland) to the machine. The time needed for transporting 250 mL air through the machine was measured to regulate the flowmeter exactly to 1 L/min. The flow was determined before every evaporation and after each experiment by attaching the syringe to the machine and measuring 15 times the gas transportation time. Finally, the reproducibility of the washing bottles was tested and compared with a standard glass washing bottle for estragole.

### Temperature-dependent evaporation of aroma compounds

Each aroma standard of 50 mg/kg was produced in 1:1 pure propylene glycol (PG) and glycerin (G), if not indicated otherwise. The evaporation was made with an E-leaf iStick TC60W e-cigarette with a 0.75 Ω iron coil. Puffs with a duration of 2 s and 28 s break between the puffs were produced. The puff session contained 10 puffs. The wash solutions of both falcons were mixed together and the solvent containing the aroma compound was measured with gas chromatography-mass spectrometry (GC/MS). For the temperature-dependent studies, each e-liquid was evaporated at six different temperatures from 105 to 315 °C in 25 °C steps. Concentrations between 1000 and 125 mg/kg aroma in the tank were produced for benzaldehyde and both pinenes to analyze the concentration dependence of tank concentration and vapor concentration at an e-cigarette temperature of 220 °C. Limonene was evaporated using following e-liquid base composition in % (m/m): PG/G = 60/40; 30/70; 60/30/10 water and an e-cigarette temperature of 220 °C. Each evaporation was produced in triplicates. For comparison of the evaporation, an ANOVA (analysis of variances) was calculated with a certainty of 95%.

### GC/MS method—liquid injection

An Agilent 6890 N gas chromatograph coupled with an Agilent 5973 inert mass spectrometer was used. An Agilent HP5-MS column (30 m, 0.25 mm ID, 0.25 μm) was applied using helium with 1 mL/min as mobile phase. One microliter was injected with a split of 1:50 and an inlet temperature of 300 °C. The temperature gradient was as follows: 60 °C hold for 3 min, 8 °C/min to 160 °C hold for 1 min, 15 °C/min to 250 °C hold for 1 min. The mass spectrometer was operated in SIM mode for each desired aroma compound: The GC/MS method was calibrated with standards of each compound in acetonitrile (see Electronic Supplementary Material (ESM) Table S1 for calibration curves, *R*^2^, LOD, and LOQ).

### Vapor sample preparation

The thermal desorption tubes were filled manually with 180 mg of different adsorbents. Standard mixtures were absorbed onto the adsorbents and measured with the optimized GC/MS method mentioned in 2.6 twice. Carbotrap B leads to the highest peak signal for each desired compound and a desorption of 100%, which is why Carbotrap B was finally used as adsorbent. The tube was connected on one side to the mouth piece of the e-cigarette and on the other side with a 50-mL glass syringe. Two e-cigarettes were used: An E-Leaf iStick TC60 Watt operated with either a 0.75 Ω nickel (E-Leaf nickel) or a 0.75 Ω iron coil (E-Leaf iron) and a Fumitech Ferobox 45TC e-cigarette coupled with a 0.75 Ω iron coil (Fumitech iron). Each e-cigarette was operated at 315 °C. For each experiment, one puff with a volume of 33 mL in 2 s was taken. For blank measurements, a 1:1 mixture of pure propylene glycol and glycerin were evaporated three times with each e-cigarette. Samples containing either 500 mg/kg limonene or 500 mg/kg linalool were evaporated three times with each e-cigarette.

### Thermal desorption GC/MS

For analysis of the oxidation of aroma compounds, the same type of GC/MS (Agilent 6890 N gas chromatograph coupled with an Agilent 5973inert mass spectrometer) equipped with the same column (Agilent HP5-MS, 30 m, 0.25 mm ID, 0.25 μm) was used. The thermal desorption unit was a Gerstel TDSA2 coupled with a CIS4. Helium with 1 mL/min was the mobile phase. The thermal desorption unit had an initial temperature of 50 °C which was hold for 1 min. It was heated at a rate of 100 °C/min to 300 °C and held for 5 min. The TDU was operated in solvent venting mode with a vent time of 1 min and a transfer temperature of 320 °C. The CIS unit used cryo cooling. Its initial temperature was 2 °C held for 1 min. With a rate of 10 °C/s the CIS unit was heated to 320 °C and held at this temperature for 5 min. The GC oven gradient was as follows: 80 °C held for 3 min, 2 to 110 °C, held for 2 min, 15 °C/min to 280 °C, held for 3 min. Solvent vent was used as split mode with a vet flow of 90 mL/min. The mass spectrometer was operated in SIM mode with the desired mass/charge values. For the determination of the limit of detection (LOD), a gas standard was produced in a 1-L gas bulb by adding 0.5 μL of each desired aroma compound (limonene oxide, carvone, carveol, linalool oxide, and p-cymene). The standards were diluted in a 2-L gasbag and measured with the mentioned GC/MS method (see ESM Table S2 for calibration curves, concentration ranges, *m*/*z* values and LOD/LOQ).

## Results and discussion

### Smoking machine

The smoking machine shown in Fig. 1 is made out of inexpensive parts and reduces the amount of solvent from 25 ml, used in typical glass wash bottles, to 4 ml. By performing only ten puffs per session, it can be operated manually. The gas flow was adjusted to 1.03 L/min (± 0.013 L/min) which varied over time only by 1.2%. Comparing the falcons as a washing bottle with a standard glass washing bottle leads to a lower CV and a higher recovery (100% compared to 90%) of estragol. The here used smoking machine showed an overall reproducibility below 20%, compared to the CVs of present literature, although the reproducibility is temperature dependent. Benzaldehyde showed a CV of 1.6% at 315 °C, whereat it lied at 10.3% at 210 °C. Estragole had similar CV behavior (CV_210°C_ = 19.2%, CV_315°C_ = 6.8%). However, α- and β-pinene showed the lowest CVs at 210 °C (CV_α-pinene_ = 3.8%, CV_β-pinene_ = 4.6%) and the highest CVs at 105 °C (CV_α-pinene_ = 9.4%, CV_β-pinene_ = 21.9%). Finding the lowest error, which can indicate high reproducibility, seems to depend from the analyzed compound.

### Evaporation behavior of the aroma compounds

#### Temperature dependence

**Fig. 2:**
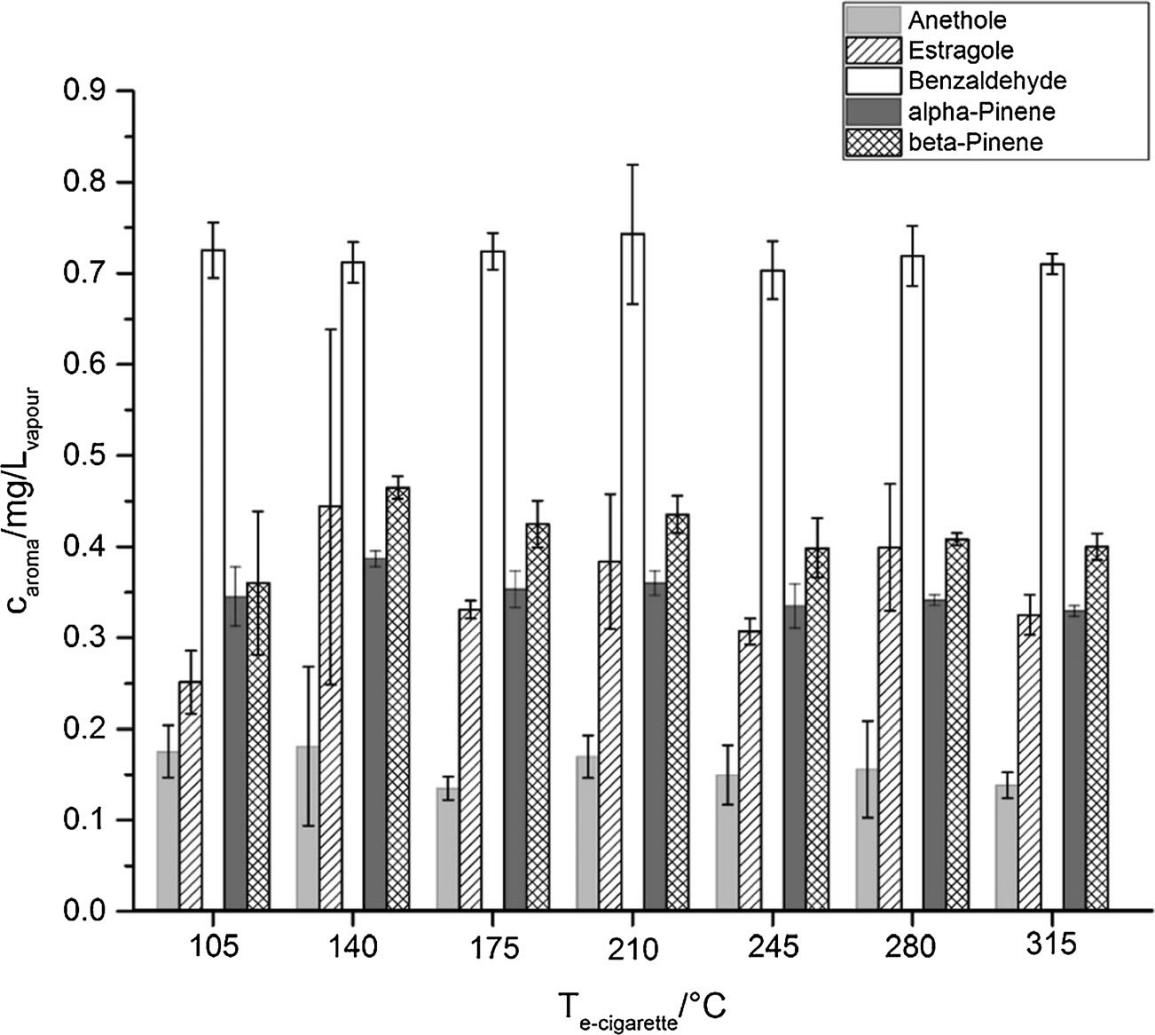
Dependence of e-cigarette temperature and concentration of aroma compound in the vapor for t-anethole, estragole, benzaldehyde, α-pinene and β-pinene

Figure 2 (and ESM Table S4) shows the amount in mg of aroma compound per liter e-cigarette vapor. Surprisingly, no temperature dependence is observed for the aroma compounds. Calculating the Pearson correlation coefficient for each compound, it lies between − 0.481 for α-pinene and 0.115 for estragole for the correlation of the e-cigarette and the aroma concentration in the vapor. β-pinene with a boiling point (bp) of 164 °C evaporates more likely as its isomer α-pinene with a bp of 155 °C. Reasons for that could be found in the higher solubility (log(o/w)_β-pinene_ = 4.16, log(o/w)_α-pinene_ = 4.83) of β-pinene in polar solvents although the vapor pressure of α-pinene (4.75 mmHg) is higher than of β-pinene (2.93 mmHg). Benzaldehyde with a bp of 178 °C and a log(o/w) of 1.48 is found in the highest concentration in the vapor. Concerning t-anethole and its isomer estragole, the latter evaporates less likely. T-anethole has not only a higher bp (235 °C) than estragole (216 °C) but also is less soluble in polar solvents (log(o/w)_t-anethole_ = 3.39, log(o/w)_estragole_ = 3.51) which could explain this behavior.

#### Base composition and aroma compound characteristic

Limonene was evaporated with different e-liquid base compositions. If the polarity of the e-liquid base is increased by adding water, the *p* value of 0.051 (*α* = 0.05) could show the low influence of water on the amount of aroma in the vapor. However, if the propylene glycol content is varied, the *p* value for the comparison of a 60/40 PG/G and 30/70 PG/G e-liquid is with 8.99·10^−4^ (*α* = 0.05) significant for an influence.

Mentioning above that the solubility affects the aroma amount more likely, 1,8-cineol (bp = 176 °C, log(o/w) = 2.74) can be compared with α-terpinene (bp = 174 °C, log(o/w) = 4.25). Both have similar bp but 1,8-cineol is more soluble in polar solvents. 1,8-cineol can be found in the vapor with a concentration of 0.37 mg/L (± 0.02 mg/L), α-terpinene occurs in lower amount in the vapor (0.300 mg/L (± 0.003)). This phenomenon is also found when comparing limonene (0.58 mg/L ± 0.04) and benzaldehyde (0.82 mg/L ± 0.02), both with similar boiling points. The ANOVA shows here a significant difference in the vapor concentration (4.4 × 10^−4^ (*α* = 0.05)).

#### Tank concentration

**Fig. 3:**
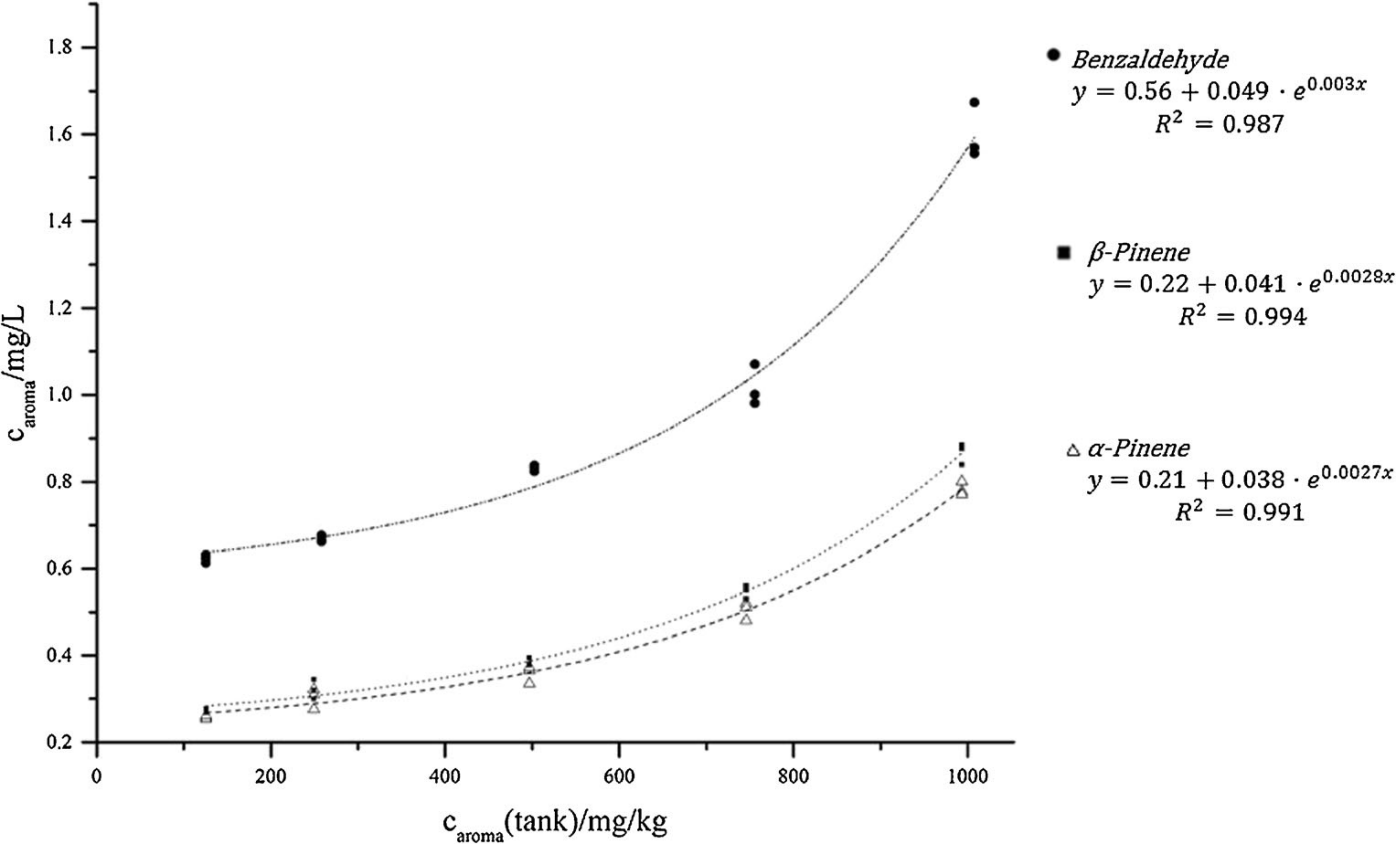
Evaporation behavior of benzaldehyde, α- and β-pinene at different tank concentrations using an E-leaf iStick TC60W e-cigarette equipped with a 0.74 Ω iron coil and a temperature of 220 °C

Different amounts of benzaldehyde, α- and β-pinene were evaporated with the same settings between a concentration of 125 and 1000 mg/kg. Figure 3 (and ESM Table S3) shows the dependence of the aroma concentration in the vapor und the tank concentration. The curve of benzaldehyde lies above those of both pinenes due to its higher solubility in the polar e-liquid base. An exponential fit was added since it gave the best coefficient of determination (*R*^2^). The information that the aroma compounds evaporate with an exponential behavior is quite important for risk assessments.

### Oxidation of limonene and linalool

**Fig. 4:**
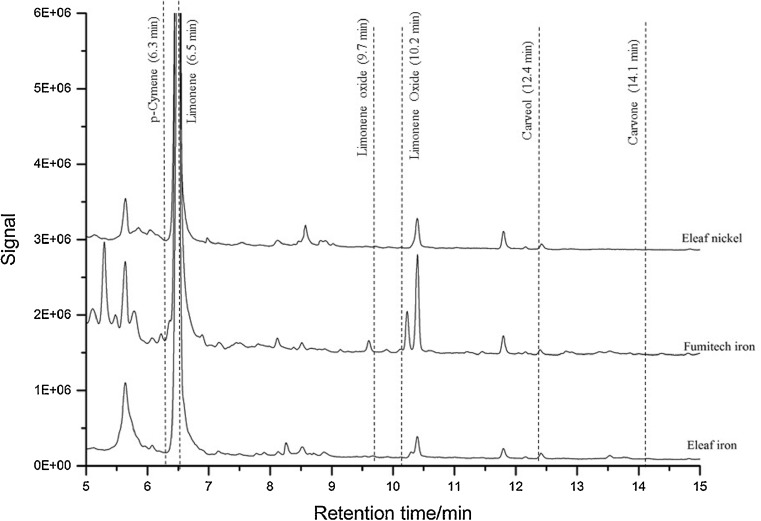
TDU measurement of limonene e-liquid (500 mg/kg) at 315 °C with two different e-cigarettes and two different atomizers. The retention times of the desired compounds are shown

**Fig. 5:**
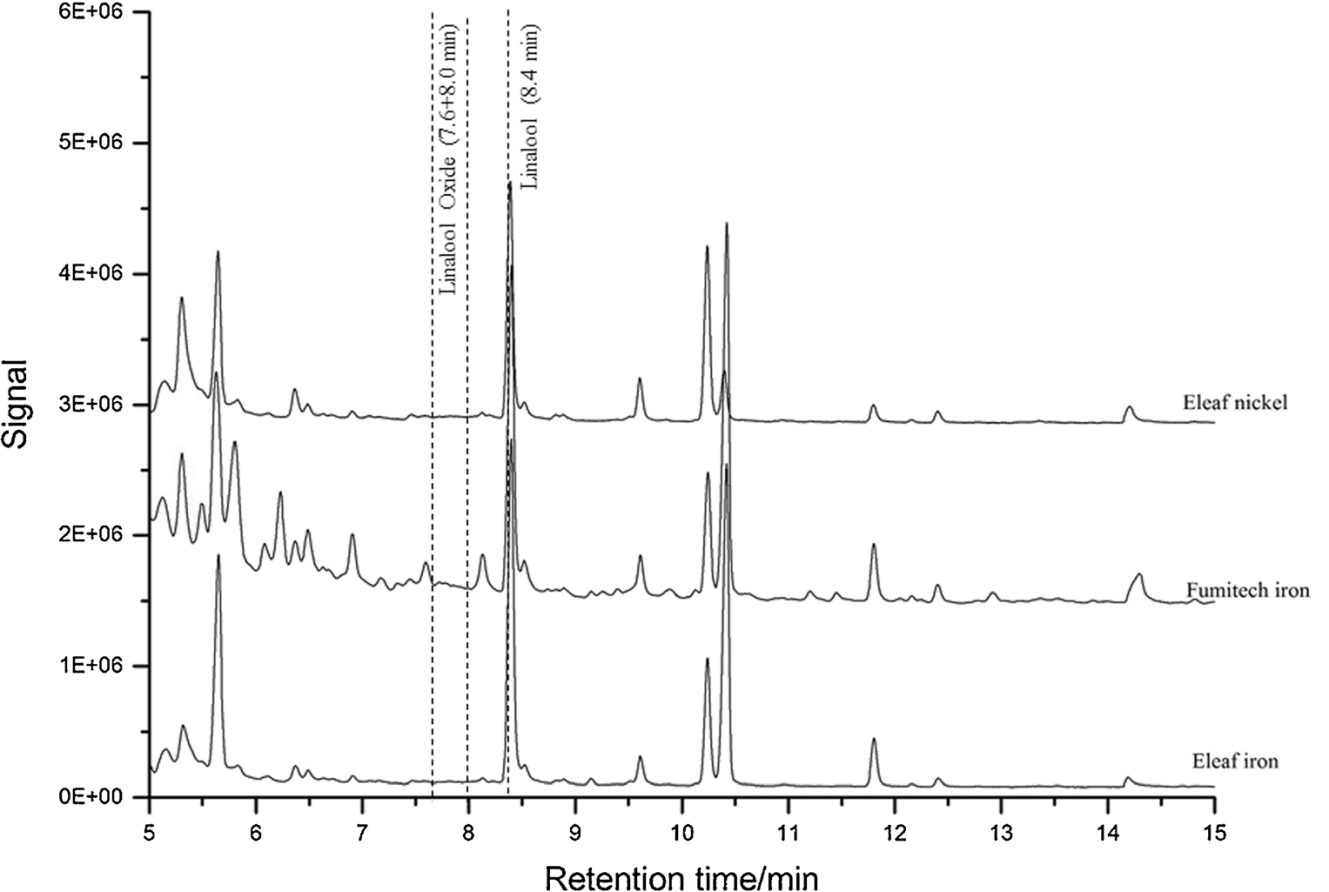
TDU measurement of linalool e-liquid (500 mg/kg) at 315 °C with two different e-cigarettes and two different atomizers. The retention times of the desired compounds are shown

Carbotrap B achieved the highest adsorption of the desired aroma compounds. The calibration parameters are found in the supplement materials. Figure 4 shows the chromatograms acquired using the e-cigarettes with different coil materials at 315 °C. No characteristic peaks for any oxidized compounds were found, which indicates that limonene does not oxidize in the e-cigarette. Additionally, the chromatograms for linalool (Fig. 5) also show no oxidation products. 315 °C is the highest adjustable temperature although consumers would not operate the e-cigarette at such a high temperature since the produced vapor smells burnt (because of the cotton in the coil, which starts to burn).

## Outlook

This work shows the behavior of aroma compounds evaporated by an e-cigarette. The analysis of the vapor was carried out using a self-made smoking machine. Its coefficients of variation are in contrast to the literature below 20%. No temperature dependence is observed for the tested compounds as demonstrated by the low correlation between temperature and concentration in the vapor. The main physical value which affects the concentration of the aroma compound in the vapor is probably the solubility in polar solvents expressed as log(o/w). It is important to know how the compounds evaporate to establish a risk assessment or a threshold value, which do not endanger the end consumer. Benzaldehyde has a LC of 0.5 mg/L air for rats, which would be achieved within 10 puffs using a 500 mg/kg benzaldehyde e-liquid. If the concentration of the aroma compound is even higher, concentration in the vapor increases exponentially, and hence, even less puffs are necessary to reach a certain critical value. It is important to know how the aroma compounds evaporate to compile a risk assessment. The data of Farsalinos [17] show that an average e-cigarette user takes 43 puffs in 20 min, whereas one puff takes 4 s. Taking the average concentration of estragole at 220 °C, after 50 puffs, 1 mg of estragole or 2.4 mg of benzaldehyde would enter the body, respectively. The next step to lower risks for the consumer would be the analysis of absorbed aroma compounds in the lungs via for example PTR-MS.

## Electronic supplementary material

ESM 1 (PDF 191 kb)
