# Supplementary material for: Investigation of the evaporation behavior of aroma compounds in e-cigarettes
Source: Anal Bioanal Chem. 2019 Mar 16;411(14):3029–35. doi: 10.1007/s00216-019-01749-7 (PMC6526143; doi:10.1007/s00216-019-01749-7)
Supplement: Supplementary file 1 — (PDF 191 kb) [file 216_2019_1749_MOESM1_ESM.pdf]

Analytical and Bioanalytical Chemistry

Electronic Supplementary Material

Investigation of the evaporation behavior of aroma compounds in e-cigarettes

Jean-Christophe Noël, Veronika Ruzsanyi, Matthias Rainer, Günther Bonn

Calculation of Accuracy: Standard in Matrix in known concentration calculated as followed:

$$Accuracy = \frac{c_{measured}}{c_{added}} \cdot 100\,\%$$

Table S1 Calibration parameters of the aroma compounds which were studied for their evaporation behavior

compound	m/z/Da	concentration range mg/kg	calibration curve	R^2	lod/mg/kg	loq/mg/kg	Accuracy/%
**Anethole**	**148.1** , 121.0	3274.0-327.4	2.29E4 · x + 6.25E4	0.9951	133.7	525.3	108
**Estragol**	**148.1** , 121.0	164.0-16.0	2.12E4 · x - 2.65E4	0.9965	6.7	27.3	96
**Benzaldehyde**	**106.1**, 77.1	326.0-13.0	1.57E4 · x - 6.07E4	0.9942	12.6	47.9	93
**Limonene**	**68.0**, 93.0	855.0-53.2	6.90E3 · x - 2.48E5	0.9910	22.5	84.2	104
**α-pinene**	**93.0**, 77.0	125.5-20.1	4.98E4 · x - 5.62E5	0.9944	2.9	10.6	103
**β-pinene**	**93.0**, 41.0	125.7-20.1	4.56E4 · x - 5.58E5	0.9937	3.0	11.2	105
**1,8-cineol**	**81.1**, 108.1	89.5-19.9	1.10E4·x-1.33E5	0.9875	6.0	23.0	99
**α-terpinene**	**93.1**, 121.2	88.5-19.9	1.39E4·x-2.14E5	0.9851	6.6	25.0	95

Table S2 Calibration parameter of the aroma compounds which we looked for in the oxidation in the e-cigarette. Limonene oxide and linalool oxide were only available as reacemic mixtures. Their calibration was done by adding both peaks

compound	m/z/Da	concentration range µg/L	calibration curve	R^2	lod/µg/L	loq/µg/L	Accuracy/%
**p-Cymene**	**119.0**, 134.0	498.6-21.5	3.77E4·x-2.05E6	0.9883	15.4	30.5	95
**Carvone**	**82.0**, 108.0	497.3-21.5	5.45E3·x-2.43E4	0.9847	17.6	34.9	93
**Carveol**	**119.0**, 91.0	142.8-21.5	67.17·x-8.6E3	0.9840	18.0	35.7	94
**Limonene Oxide**	**67.0**, 94.0	474-20.5	1.66E3·x-7.24E4	0.9853	16.5	32.7	95
**Linalool Oxide**	**59.0**, 94.0	431.9-18.6	3.20E3·x-2.54E5	0.9819	16.7	33.1	98

Table S3 Mean concentration and standard deviation of data representing in Fig. 3

Benzaldehyde		α-Pinene		β-Pinene	
c(Tank)mg/kg	cVapour/mg/L	c(Tank)/mg/kg	c(Vapour)/mg/L	c(Tank)/mg/kg	c(Vapour)/μg/L
125.69	0.61	125.67	0.26	125.67	0.27
125.69	0.62	125.67	0.25	125.67	0.27
125.69	0.63	125.67	0.26	125.67	0.28
257.81	0.68	249.33	0.31	249.33	0.34
257.81	0.67	249.00	0.32	249.00	0.30
257.81	0.66	249.33	0.28	249.33	0.32
502.51	0.82	496.90	0.37	496.90	0.39
502.51	0.83	496.90	0.33	496.90	0.38
502.51	0.84	496.90	0.37	496.90	0.38
755.68	1.00	745.59	0.52	745.59	0.55
755.68	1.07	745.59	0.51	745.59	0.56
755.68	0.98	745.59	0.48	745.59	0.53
1008.00	1.57	993.27	0.78	993.27	0.88
1008.00	1.67	993.27	0.77	993.27	0.84
1008.00	1.56	993.27	0.80	993.27	0.88

Table S4 Mean concentrations and standard deviations for data represented in Fig. 2

	Anethole		Estragol		Benzaldehyde		α-Pinene		β-Pinene	
T/°C	c(mean)/mg/L	±s/mg/L	c(mean)/mg/L	±s/mg/L	c(mean)/mg/L	±s/mg/L	c(mean)/mg/L	±s/mg/L	c(mean)/mg/L	±s/mg/L
105	0.175	0.028	0.251	0.035	0.725	0.031	0.345	0.032	0.360	0.079
140	0.181	0.087	0.444	0.195	0.712	0.022	0.387	0.009	0.465	0.012
175	0.135	0.013	0.331	0.010	0.724	0.020	0.353	0.020	0.425	0.025
210	0.170	0.023	0.384	0.074	0.743	0.077	0.360	0.014	0.435	0.020
245	0.150	0.033	0.307	0.014	0.703	0.032	0.335	0.024	0.399	0.032
280	0.156	0.053	0.399	0.069	0.719	0.033	0.342	0.006	0.408	0.007
315	0.138	0.014	0.325	0.022	0.710	0.011	0.329	0.006	0.400	0.015
